# Outcome Analysis of Dual Plating in Management of Unstable Bicondylar Tibial Plateau Fracture - A Prospective Study

**DOI:** 10.5704/MOJ.2111.005

**Published:** 2021-11

**Authors:** M Raj, SPS Gill, A Rajput, KS Singh, KS Verma

**Affiliations:** 1 Department of Orthopaedics, All India Institute of Medical Sciences, Deoghar, India; 2 Department of Orthopaedics, Uttar Pradesh University of Medical Sciences, Etawah, India

**Keywords:** unstable bicondylar tibial plateau fracture, Schatzker type V and VI, Rasmussen’s criteria, oxford knee score, radiological outcome

## Abstract

**Introduction:**

Bicondylar tibial plateau fractures account for 10-30% of tibial plateau fractures. Despite recent advancements in the management of unstable bicondylar tibial plateau fractures, the outcomes are often poor. The present study aimed to evaluate the functional outcomes and complications of internal fixation of bicondylar tibial plateau fractures with the dual plating using two incisions.

**Materials and methods:**

The present study included 30 patients (26 males; 4 females, mean age 35.6 years; range, 19 to 65 years) with bicondylar tibial plateau fractures who were treated with dual plating between January 2017 to August 2019. Out of 30 patients, 5 patients had Schatzker type (V) and 25 patients had Schatzker type (VI) bicondylar tibial plateau fracture. All patients were treated with dual plating using two incisions. In all patient’s similar standard physical rehabilitation therapy was followed. All complications including intra and post-operative were assessed and recorded. The patients were followed-up for over 24 months. Functional outcomes were assessed with Rasmussen’s functional grading system, Oxford knee score, and range of motion of knee joint. Radiological outcomes were evaluated using Rasmussen’s radiological scoring system.

**Result:**

All fractures united with a mean time of 18 weeks. The average knee range of motion was 1.5° - 130° (range: 0° - 10° for extension lag, range: 100° -135° for flexion). Mean Rasmussen's functional grading score at the final follow-up was 26.75. All patients showed excellent or good radiographic results according to Rasmussen’s radiological scoring with a mean score of 8.5 (range 6-10). The post-operative radiographs showed mean MPTA was 84.3° and the mean PPTA was 6.2°. In the present study, complications were encountered in five patients. However, there were no cases of secondary loss of reduction, failure of the implant, malunion, or non-union.

**Conclusion:**

The surgical treatment of bicondylar tibial plateau fractures with dual locking represents a significant treatment option and provides rigid fixation in these fractures with good functional and radiological outcomes.

## Introduction

Tibial plateau fractures represent a wide spectrum of fractures that ranges from simple injuries to complex fracture patterns and are the result of a very high energy impact between bone and another surface. According to Schatzker classification, type V, VI are unstable tibial plateau fractures which include bicondylar injuries with significant articular depression, multiple displaced condylar fracture lines, meta-diaphyseal fracture extensions or comminutions, with the significant compromise of the soft tissues envelope^1,2^. The treatment strategy of these fractures includes the preservation of soft tissues, reduction of articular congruity, and maintenance of anatomic alignment in the lower extremities with a good range of functional outcomes^3^. However, the treatment of these fractures was thought to be prognostic of poorer clinical outcomes and remains a challenging task for the treating surgeon. If we look through the literature, many treatment modalities have been proposed. Non-operative management of bicondylar fractures of the proximal tibia by traction and cast was very common in earlier decades but stiffness and malunion were the main complications of non-operative management^4^. Traditional techniques of external fixation or hybrid fixator as a means of realigning the metaphysio-diaphyseal anatomy gained some popularity, however, it was associated with its unique complications such as the potential for septic arthritis of the knee, the risk of pin site infection, and poor patient compliance^5,6^. Modern instrumentation such as a locking plate system and newer techniques of internal fixation like MIPO (minimally invasive plate osteosynthesis) had changed the nature of the treatment of these high-energy tibial plateau fractures.

A single lateral locking plate provides adequate stability and can be an effective implant in cases of bicondylar tibial plateau fractures. It avoids stripping of soft tissue for the additional medial plate with limited dissection and percutaneous insertion of locking screws through the guide arms, which further reduces the soft tissue insult at the time of surgery^7^. However, maintenance of axial alignment is difficult in cases of unstable bicondylar fractures with a single lateral plate fixation especially in cases, where the articular component of the medial column fracture had a coronal fracture line^7,8^.

Stabilising both medial and lateral columns through two-incision dual plating has been preferred fixation for bicondylar tibial fractures. Double incision provides better visualisation of fracture geometry intra-operatively and dual plating provides rigid fixation of both tibial condyles preventing medial collapse and subsequent varus collapse^9,10^. However, two different incision results in extensive soft tissue dissection and periosteal injury leading to a higher incidence of wound complications^11^. This study was planned to determine the clinical and radiological outcomes in bicondylar tibial plateau fracture managed by dual plating with a two-incision technique and the complications associated with the procedure.

## Materials and Methods

We conducted a hospital-based descriptive study from January 2017 to August 2019. A total of 30 patients which includes 26 males and 4 females with bicondylar tibial plateau fractures were managed with dual plating. The mean age was 35.6 years ranging from 19 to 65 years. Inclusion criteria include skeletally matured patients above 18 years of age presented with unstable bicondylar tibial plateau fractures i.e. Schatzker’s type V, VI. Exclusion criteria include Gustilo open fracture grade 2 or 3, tibial plateau fracture of type (1-4) according to Schatzker’s classification, pathological fractures, patients presented with severe systemic illness or medical conditions not suitable for surgery. The mechanism of injury were road traffic accidents in 26 patients and others were falls from a height or to the ground. Demographic details of patients are shown in Table I.

**Table I:** Patient demographic details

S.No.	Variable	Demography
1	Total number of Patients	30
2	Mean Age (in years) Range	35.6 (19-65)
3	Male : Female	26/4
4	Mode of Injury	
	Traffic Injury	26
	Fall	4
5	Side Involvement	
	Left	11
	Right	9
6	Fracture Type (Schatzker)	
	Type V	5
	Type VI	25
7	Associated Fracture	7
8	Duration of follow-up (in months)	22.4 (14 ^⁓^ 48)

On admission, the patients were assessed clinically. The injured extremity was examined to define the personality of fracture with particular attention to swelling, neurovascular injury, skin condition, joint effusion, and shortening of the limb. The standard anteroposterior and lateral radiographs were taken to assess tibial plateau fractures. In cases where fracture geometry could not be adequately assessed then a CT scan with 3-D reconstructions was done. All tibial plateau fractures were classified based on Schatzker classification. Schatzker Type V and Type VI fractures with tense knee joint effusion, knee aspiration was performed under aseptic condition. Calcaneal or lower tibial skeletal traction was applied and the limb was kept elevated on Bohler Brown frame till the tissue edema settled (wrinkle sign appeared) and skin condition became good enough to post the patient for surgery. Three Senior orthopaedic surgeons from the same unit in our hospital (each having exposure of more than seven years) had performed the surgery.

In all patients, the operation was performed in a supine position under spinal anaesthesia using a pneumatic tourniquet with a folded pillow under the knee. Under the C arm image intensifier after proper soft tissue dissections, joint line congruity was checked first. Percutaneous K wires were used to hold the fragments in reduction. Special attention was placed on the articular depression if present, a bone punch was used to elevate the depressed portion and the void was filled with bone graft. If needed, a sub-meniscal view through lateral incision was used to achieve anatomic reduction. We typically fixed the medial tibial condyle first. Through the posteromedial approach, the medial tibial condyle fracture fragment visualised, reduced, and fixed with 4.5mm proximal tibial T or L buttress plate or screw s after contouring. Through the Anterolateral approach, lateral condyle tibial fracture was reduced and fixed with a proximal tibial lateral locking compression plate.

In all the surgeries wounds were closed over suction drains. The drains were removed after 48 hrs. On the 3rd post-operative day, isometric quadriceps exercises and knee range of motion were initiated depending upon the fracture geometry and stability of the fixation. Patients were kept on non-weight bearing for a minimum of 10 weeks. Partial weight-bearing was started from 10-14 weeks depending upon the fracture configuration and correlation with the radiograph. Full weight-bearing is allowed only after the radiological healing of the fracture. Bony union was defined radiographically when at least three cortices united.

Patients were called for follow-up weekly for one month, then monthly for three months, and thereafter at six months. Patients were examined for skin condition including signs of infection, range of movement of the knee, implant failure, loss of reduction, or any other complication related to the union or soft tissue. Functional outcomes were assessed with Rasmussen’s functional grading system, Oxford knee score, and range of motion of knee joint.

**Fig. 1:**
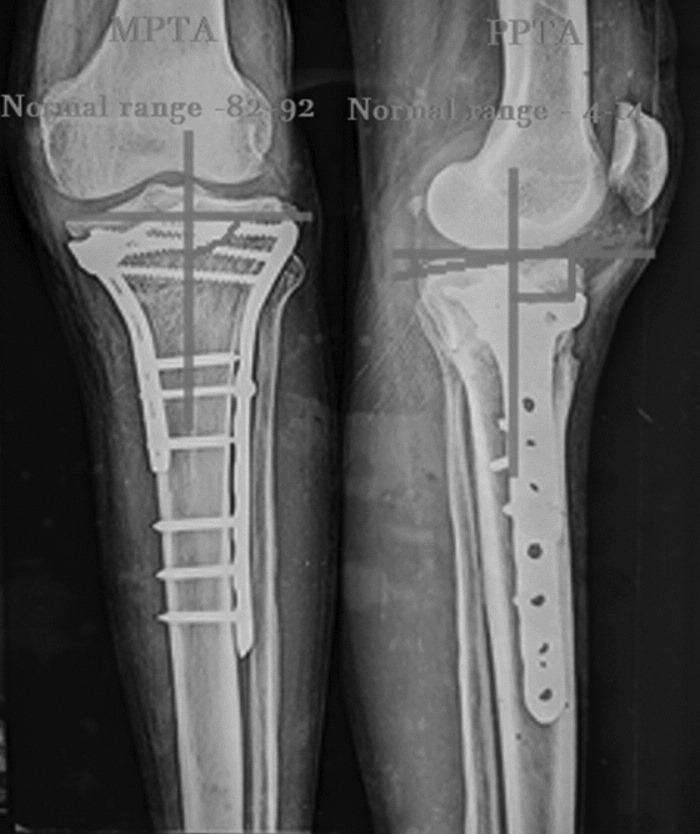
Radiographic measurements of MPTA and PPTA angle.

**Fig. 2:**
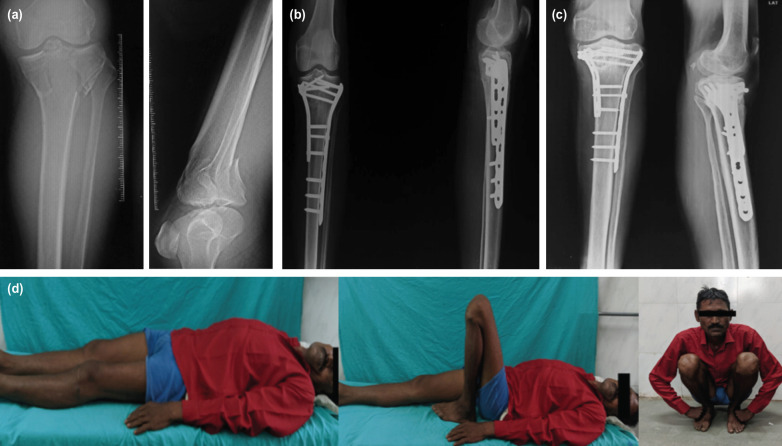
(a) Radiograph showing bicondylar tibial plateau fracture. (b) Post-operative radiograph showing fixation with dual plating. (c) Six months post-operative radiograph showing union. (d) Clinical pictures showing good range of movement.

Outcomes were evaluated using Rasmussen’s radiological scoring system. The radiological union was described as the presence of callus formation in 3 out of 4 cortices. Post-operative tibial plateau fracture reduction status and alignment of the proximal tibia were assessed both in the sagittal and coronal plane through the Posterior proximal tibial angle (PPTA) and Medial proximal tibial angle, respectively. MPTA is the medial angle between the tangential line and anatomical axis of the tibia in AP radiographs (Fig. 1) (normal range: 82° - 92°). PPTA is the angle between the tangential line of the medial plateau and the perpendicular line of the anterior tibial cortex on lateral radiographs. (normal range: 4° - 14°). Radiological assessment was done from immediately after surgery till the last visit at the final follow-up. An increase of 5° malalignment or an articular depression of 2mm compared with the first post-operative radiograph was defined as a secondary loss of reduction. Data analysis was conducted using SPSS software (version 20). Descriptive method of analysis was used for analysis and interpretation of results (Fig. 2).

## Results

In the present study, 30 patients with tibial plateau fractures fulfil the inclusion criteria. The mean follow-up time was 22.4 months (range 14-48). 5 patients had Schatzker type (Ⅴ) and 25 patients had Schatzker type (Ⅵ) bicondylar tibial plateau fracture. The average duration between injury and definitive surgery was 8.9 days (range 4-16 days). The mean duration of surgery was 80.67±7.28 minutes. The average duration of hospital stay was 12.7 days (range 10-30). Primary bone grafting was done in four patients. All fractures united with a mean time of 18 weeks. The average time to unprotected full weight-bearing was 18.9 weeks (range 14-22). Table II shows perioperative and post-operative patient details. The average knee range of motion was 1.5° - 130° (range: 0° - 10° for extension lag, range: 100° - 135° for flexion). Mean Rasmussen’s functional grading score at the final follow-up was 26.75. Out of 30 patients, 15 (50%) patients had an excellent result with an average score of 28, 12 (40%) patients had good results with an average score of 26.50, 2 (6.67%) patients had fair results with an average score of 21.50 and 1 (3.33%) patients had a poor result with an average score of 19. The mean Oxford knee score at the final follow-up was 43.24±3.5. All patients showed excellent or good radiographic results according to Rasmussen’s radiological scoring with a mean score of 8.5 (range 6-10). The post-operative radiographs showed a mean MPTA was 84.3 and the mean PPTA was 6.2° in the present study. However, we cannot achieve any statistically significant difference between the operated knee and normal uninjured knee (p=0.43 for MPTA, p=0.64 for PPTA). Table III shows post-operative functional scores and radiological outcomes.

**Table II:** Perioperative and post-operative patient details

S.No.	Variable	Parameter
1	Primary/Staged	25/5
2	Pre-surgical stay (in days)	8.9 (4 ⁓ 16)
3	Duration of surgery (in minutes)	80.67±7.28 (60 ⁓ 180)
4	Blood loss (in ml)	144.6 (80 ⁓ 340)
5	Duration of hospital stay (in days)	12.7 (10 ⁓ 30)
6	Weight-bearing (in weeks)	13.8 (10 ⁓ 24)
7	Primary bone grafting	4

**Table III:** Post-operative functional scores and radiological outcome of patients

S.No.	Variable	Parameter
1	Range of Motion	
	≥ 120°	23
	≤ 120°	7
2	Oxford Knee Score (OKS)	43.24 (34 - 45)
3	Rasmussen’s functional score (RFS)	23.75 (19 - 28)
4	Post-operative medial proximal tibial angle (MPTA) (At final follow-up)	84.3 (82 - 91)
5	Post-operative Proximal posterior tibial angle (PPTA)	6.2 (4 - 11)
6	Rasmussen’s radiological score (RRS)	7.86 (6 - 10)

In the present study, complications were encountered in five patients. Superficial infection was found in two patients. However, the culture report showed no growth of the organism. Empirical antimicrobial therapy was given for the next two weeks and the superficial infection healed subsequently. One patient presented with a deep infection for which debridement and posteromedial plate removal were done. Other complications include the presence of articular depression in one patient and varus collapse in one patient. Late complication includes the presence of flexion contracture of knee joint (<10°) in one patient which was managed with knee mobilisation exercises. There were no cases of secondary loss of reduction, failure of the implant, malunion, or non-union. Table IV shows post-operative complications encountered in the present study.

**Table IV:** Post-operative complications

S.No.	Variable	Complication
1	Infection	
	a) Superficial	3
	b) Deep	1
2	Radiological Malunion	
	a) Articular Depression	1
	b) Malalignment	1
	c) Secondary loss of reduction	0
	d) Failure of Implant/ Loosening of Screw	0
3	Stiffness	1

## Discussion

High energy bicondylar tibial plateau fractures (Schatzker type V, VI) are unstable intraarticular fractures whose treatment has always remain a challenge to treating surgeons. The aim of the operative treatment of these fractures includes anatomic reduction of fracture fragments, maintenance of articular congruity, preservation of surrounding soft tissues, and avoidance of complications, particularly infection and malalignment. Various method of fixation of these fractures mentioned in literature includes an external fixation, hybrid external fixation, single lateral locking plate, and dual buttress plate fixation. The ideal surgical fixation technique for tibial plateau fracture has always remained a matter of debate.

A single lateral locking plate with screws through the plate holding medial fragment can support the medial plateau and prevent varus collapse of bicondylar tibial plateau fractures. The advantage of single plating includes limited dissection and insertion of percutaneous screws through guide arms which reduces the risk of soft tissue injury and wound infection^12^. However, a single lateral locking plate has its limitations and cannot provide adequate stability and fixation in all bicondylar tibial plateau fractures. Failure of a single locking plate has been reported in cases of tibial plateau fractures having one of the following conditions or collection of them: Medial intraarticular fracture line, small comminuted medial plateau fragment, and medial articular fracture having a coronal component with posteromedial fragment^7^. Barei *et al* and Higgins *et al* reported that the incidence rate of posteromedial fragments in patients with bicondylar tibial plateau fractures was 28.8-59%^13,14^. These posterior medial fracture fragment prevents posterior subluxation of femur condyle. Single fixed angle locking plate applied laterally may not effectively engage the posteromedial fragment making reduction and fixation of complex plateau fractures difficult. Even if locking screws through the lateral plate engage the posteromedial fragment, the quality of the fixation may be insufficient to neutralise the force of displacement^7,15^. In cases of tibial plateau fractures with coronal medial fracture lines, due to the design of the locking plate, the direction of the locked screw is fixed which is parallel rather than perpendicular to a coronal fracture line^7^.

Schatzker type V and VI fractures require reduction and stabilisation of both medial and lateral condyles of the tibia. The dual plate technique fixes both medial and lateral columns and restores mechanical stability with adequate fixation^16-21^. Dual plate fixation had better biomechanical strength and less subsidence rate compared to a single lateral locking plate. Higgins *et al* performed a biomechanical study on cadavers and found that dual plate fixation allows less subsidence than the single lateral plate in bicondylar tibial plateau fracture^22^. Barei *et al* and Yoo *et al* advocated dual plate fixation and found satisfactory functional outcomes in complex tibial plateau fractures^13,23^. Malalignment post-operatively has also been reported by different authors. Neogi *et al* in their series reported post-operative malreduction and malalignment in their series and found the incidence of malalignment in 3 cases (10.9%) in the SP group and 2 cases (6.2%) in the DP group^24^. In our study malalignment with delayed loss, post-operatively occurred in 1 case (3.33%) in which varus collapse being the reason for the change in alignment. However, the functional assessment outcome was good in this patient at the final follow-up. Malreduction on immediate post-operatively was found in 1 patient (3.33%) in our study due to articular depression showing step or gap of >2mm.

The drawback of fixation with dual plating is extensive soft tissue dissections which may increase the risk of wound complications. The incidence of deep wound infection in cases with dual plate fixation was reported to be 4.7 -8.4% by different authors. Patil *et al* reported superficial wound infection in 1 patient (2.7%) with dual plating^8^. Neogi *et al* had a deep infection rate of 3.12% which was comparable to 3.33% seen in our study^24^. Superficial infection was found in 2 patients (6.66%) who healed with prolonging antimicrobial therapy. Infection is the most common complication associated with dual plating which could be avoided by gentle handling and waiting for 5-6 days after an injury so that tissue edema could subside and skin condition could improve. Table V shows a comparative analysis between the present study and other studies in the literature.

**Table V:** Comparison between the present study and other studies

	Present study	Cho KY *et al*^21^	Prasad *et al*^17^	Oh CW *et al*^20^	Citak *et al* (DP group)^10^
Type of study	Prospective	Retrospective	Retrospective	Prospective	Retrospective
Total number of patients	30	10	40	23	10
Mean age (in years)	35.6	51.6	40	54	51.2
Mean follow-up duration (in weeks)	22.4	33.7	48	25	27.8
Weight bearing (in weeks)	13.8	8	12	15	12
Range of Motion	1.5° - 130°	0° - 122.5°	1.75° - 128.5°	0° - 123°	0° - 119°
Knee score	RFS – 23.75		OKS		
	OKS – 43.24	AKSFS – 85	Excellent -30	RFS -26	RFS -22.9
			Good -10		
Wound complications	4	1	2	1	1
Malalignment	1	0	1	2	0
Secondary loss of reduction	0,	0	4	0	0
Healing Time (in weeks)	18	16	14	19	14
Mean MPTA (at final visit)	84.3°	90.5°	84.05°	Not measured	86.3°
Mean PPTA (at final visit)	6.2°	4.4°	8.25°	Not measured	5.5°

Abbreviations - RFS: Rasmussen’s functional score, OKS: Oxford knee score, AKSFS: American knee society function score, MPTA: Medial proximal tibial angle, PPTA: Posterior proximal tibial angle

This prospective study was conducted in a small cohort (n=30) of patients with bicondylar tibial plateau fractures (Schatzker’s type V and VI). Patient demographic and fracture pattern characteristics were random and not specified by the protocol. The main limitation of our series was a small number of patients, shorter duration of follow-up, and lack of a control group. We recommend large sample size randomised controlled trials with a longer duration of follow-up to devise a standard treatment protocol for the management of unstable bicondylar tibial plateau fractures.

## Conclusion

Dual plating represents a treatment option in the treatment of unstable complicated bicondylar tibial plateau fractures with stable fixation and acceptable functional results with low complication rates.
